# A Seat Belt Injury Causing a Large Breast Hematoma: A Case Report

**DOI:** 10.7759/cureus.35440

**Published:** 2023-02-25

**Authors:** Shunki Yamamoto, Yoshinori Kosaki, Takenori Uehara, Hiromichi Naito, Atsunori Nakao

**Affiliations:** 1 Department of Emergency, Critical Care, and Disaster Medicine, Okayama University Graduate School of Medicine, Dentistry and Pharmaceutical Sciences, Okayama, JPN

**Keywords:** case report, breast hematoma, chest injury, seat belt injury, traffic injury

## Abstract

Seat belts with shoulder restraints have decreased the frequency of life-threatening severe chest trauma caused by car accidents. However, the introduction of seat belt legislation has led to an increase in a specific pattern of blunt trauma known as seat belt syndrome, which includes rib, clavicle, spine, and sternum fractures, as well as rupture of hollow pelvic and abdominal viscera, mesenteric tears, and major vessel injuries. The shoulder restraint part of the three-point seat belt commonly rests near or over the female and male breast. A 54-year-old female presented to our emergency department complaining of swelling and pain in her left breast immediately after a traffic accident. The patient had used a seat belt with a shoulder restraint. Bruising was noted along her chest where there had been seat belt contact. Her breast hematoma was most likely caused by breast tissue compression between her rib and the seat belt. Contrast-enhanced computed tomography demonstrated a sizable breast hematoma with active arterial contrast material extravasation, as well as multiple left rib fractures. The patient was conservatively treated with analgesic and anti-inflammatory drugs. Complete resolution was achieved, and her breast returned to its normal appearance. Although endovascular treatment and surgical hemostasis have been proposed for the treatment of breast injuries with active bleeding, conservative treatment such as compression hemostasis may be feasible.

## Introduction

Seat belts with shoulder restraints have decreased the frequency of life-threatening severe chest trauma caused by car accidents. However, a specific type of injury has surfaced, namely, seat-belt syndrome, which includes rib, clavicle, spine, and sternum fractures, as well as rupture of hollow pelvic and abdominal viscera, mesenteric tears, and major vessel injuries. These types of injuries are often associated with soft-tissue injuries along the line of the seatbelt [1-3].

We report the case of a 54-year-old woman who developed a contusion in her left breast from a three-point seat belt following a motor vehicle accident. As the shoulder restraint part of the three-point seat belt commonly rests near or over the female breast, emergency physicians should be aware of the pathophysiology of breast trauma associated with hematoma and contusion such as that in our patient and familiarize themselves with breast trauma treatment options [4,5]. In a review of 5,305 women with blunt chest trauma, only 108 (2%) presented with breast trauma [6]. Although patients with breast hematoma with a lump or structural change sometimes visit clinics, this type of breast-related emergency must be appropriately managed and followed up long-term. We believe that sharing this case would be instructive and informative for emergency physicians for its educational value.

Written informed consent was obtained from the patient for the publication of this case report and accompanying images.

## Case presentation

**Figure 1:**
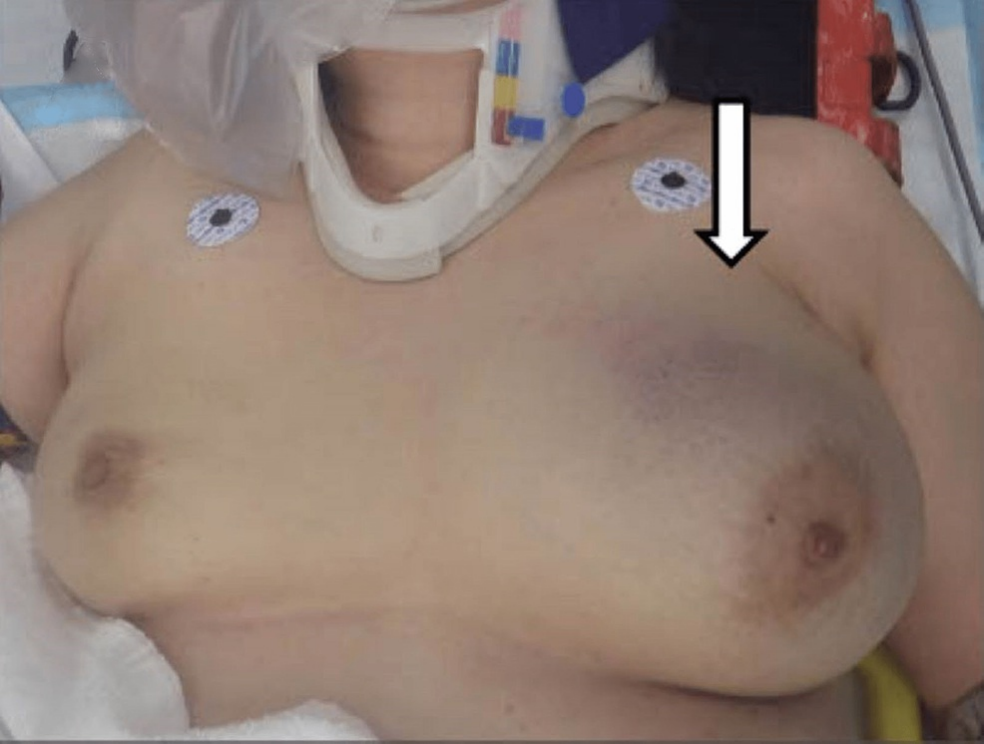
Appearance of breast hematoma. The hematoma is seen in the arrowhead area.

**Figure 2:**
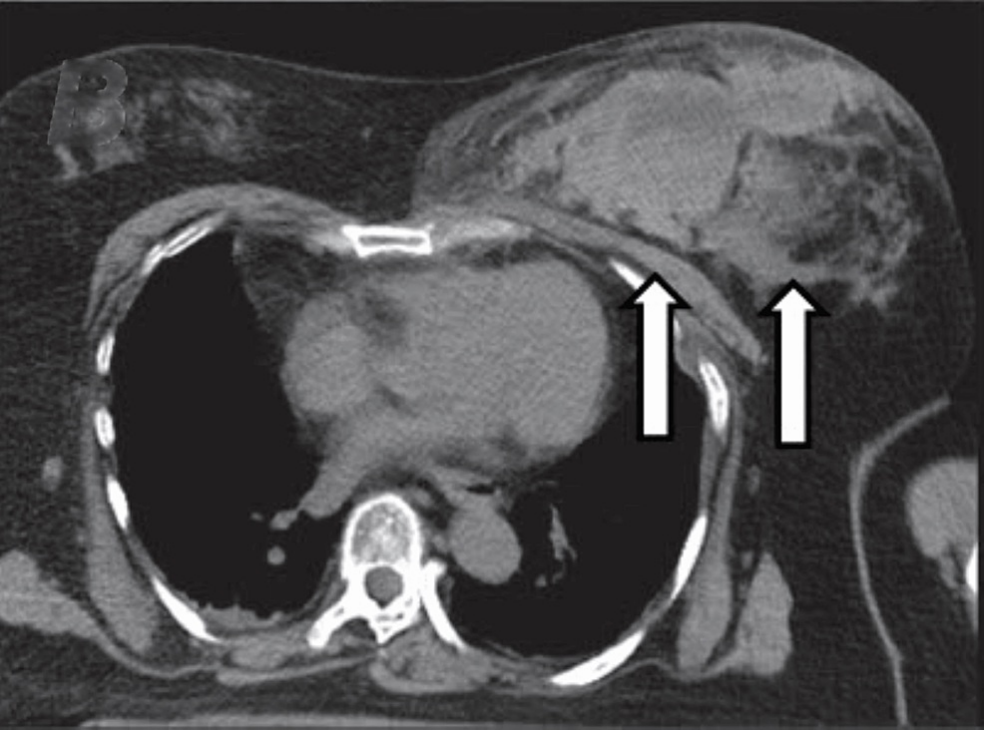
Plain computed tomography image. Plain computed tomography image of breast hematoma. A large breast hematoma is seen in the arrowhead area.

**Figure 3:**
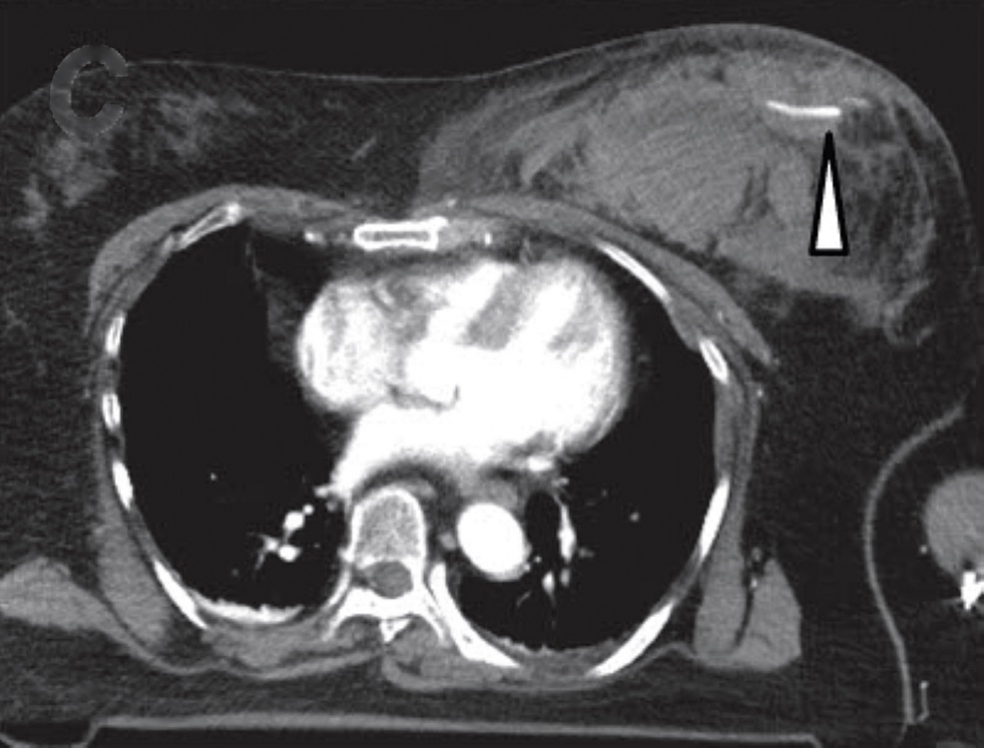
Contrast-enhanced computed tomography image. Contrast-enhanced computed tomography image of breast hematoma. Active arterial contrast material extravasation is noted in the arrowhead area.

A 54-year-old female driver was struck head-on by a car traveling at 100 km/hour and transported to our emergency department within 40 minutes. The patient was alert with stable vital signs. Her past medical history, medication history, and laboratory test results were unremarkable. She was certain that there was no breast mass before the accident. At the time of the collision, she had been wearing a three-point seat belt. Her left breast was asymmetrically enlarged and swollen with severe tenderness. Chest bruising along the seat belt line was noted (Figure 1). Plain and contrast-enhanced computed tomography (CT) of the chest and abdomen showed a large breast hematoma with active arterial contrast material extravasation, as well as multiple left rib fractures (Figures 2, 3). After consultation with the breast surgeon and radiologist, the patient was treated with compression hemostasis with a breast band, 2 g tranexamic acid, and symptomatic therapy with loxoprofen and acetaminophen. Because contrast-enhanced CT performed on the next day showed no active bleeding, no additional intervention was required. The patient was hemodynamically stable throughout the admission and was discharged on day three. Complete resolution was achieved, and her breast returned to its normal appearance after six weeks of follow-up in the breast clinic.

## Discussion

A wide range of injuries resulting from blunt breast trauma including breast soft-tissue injuries due to seat belts has been reported. In the present case, the mechanism causing the breast injury could be explained by breast tissue compression between the seat belt and bony thorax combined with sudden torso deceleration and simultaneous shearing stresses incurred by the soft tissues due to trunk rotation [7].

Some classification systems for three-point seatbelt breast trauma have been proposed. According to Majeski, there are four classifications ranging from class 1 (mild) to class 4 (avulsion breast injury) [1,4,8]. Song et al. proposed a new classification based on the time from injury to consultation and the presence or absence of shape change, which they classified into the following four categories: 1a (immediate presentation with bruising and pain), 1b (immediate presentation with expanding breast), 2a (late presentation with tender breasts, mild bruising, or palpable lump), and 2b (late presentation with structural distortion) [9]. According to Majeski’s classification, the proposed treatment for moderate crush injury (class 2) and severe crush injury (class 3) consist of analgesia and symptomatic treatment. If the hematoma is expanding, ultrasound-guided drainage may be considered. Because our patient presented active bleeding evidenced by extravasation of the contrast medium, our case was categorized as class 4. Endovascular treatment or hemostatic surgery has been suggested for the management of class 4 breast injuries, but our patient, despite continued bleeding, had stable hemodynamics and was treated conservatively with compression using a chest band with careful follow-up. A similar case treated with symptomatic therapy was reported by Teh et al. [10]. We present the cases of breast injury with active bleeding reported during 2000-2022 in Table 1 [7,10-14].

**Table 1:** Twelve patients classified in Majeski class 4 and Teo and Song class 1b.

Citation	Country	Majeski class	Teo and Song class	Diagnosis	Management
Myhre et al., 2002 [7]	USA	4	1b	Expanding breast	Arterial embolization
Teh et al., 2020 [10]	Australia	4	1b	Unspecified	Compression hemostasis
		4	1b	Unspecified	Compression hemostasis
		4	1b	Unspecified	Compression hemostasis
		4	1b	Unspecified	Symptomatic care
		4	1b	Unspecified	Symptomatic care
		4	1b	Unspecified	Symptomatic care
Yoon et al., 2014 [11]	USA	4	1b	Expanding breast	Arterial embolization
Mac New et al., 2009 [12]	USA	4	1b	Expanding breast	Arterial embolization
Patel et al., 2009 [13]	USA	4	1b	Expanding breast	Arterial embolization
Kadoya et al., 2022 [14]	Japan	4	1b	Subcutaneous hemorrhage	Arterial embolization
		4	1b	Subcutaneous hemorrhage and purpura	Arterial embolization

Endovascular or surgical hemostasis has been recommended for active bleeding, but recently there have been reports of conservative treatment of active bleeding. We consider that endovascular treatment or surgical hemostasis is highly necessary in the following cases: unstable circulation with multiple or severe trauma, severe pain; respiratory depression caused by local compression; abnormal skin findings due to worsening local circulation caused by breast swelling [14]; or when there are significant coagulation abnormalities. If none of the above conditions apply, we suggest conservative therapy, such as compression hemostasis under monitoring, as the first step for class 4 cases. Packing and surgical drainage are usually required for infected hematomas, but some can be treated with systemic antibiotics and percutaneous drainage [15]. Inflammatory reactions to the region of hemorrhage and crushed tissue consequently result in a mass pathologically known as fat necrosis [5,16].

Breast tenderness and bruising may last from weeks to months. Careful follow-up by a breast specialist is required. The firmness and irregular mass seen in the breast with blunt trauma would make it difficult to differentiate from carcinoma. Of note, there are patients who are found to have cancer in a breast damaged by trauma. Although isolated patients with skin cancer of the breast in scars are reported, there are no conclusive reports of adenocarcinoma of the breast caused by trauma. As both breast carcinoma and traffic accident are common, coincidence is the most likely explanation for this phenomenon.

Plastic surgeons may be consulted for breast deformity caused by seat belt restraint. The vast differences in cases and the need for empirical designs based on existing skin scars, estimation of breast and nipple segment blood supply, and mobility of the remaining breast are challenging for plastic surgeons [17]. Although this type of injury does not occur often, changes in seat belt design with wider belts to spread the forces incurred in accidents or additional straps to prevent rotation of the body might reduce the number of breast injuries. In the future, additional seat belt strap padding may be helpful, especially for women with large breasts.

## Conclusions

This case shows that blunt breast trauma may cause active bleeding, but if hemodynamics is stable, it can be cured with conservative treatment such as compression hemostasis. Endovascular treatment or surgical hemostasis has been recommended for breast injuries with active bleeding classified as Majeski class 4, but if the general condition is stable, it may be possible to treat without invasive treatment. Further research is expected on the treatment of breast injuries classified as Majeski class 4.
